# Premonitory Awareness Facilitates Tic Suppression: Subscales of the Premonitory Urge for Tics Scale and a New Self-Report Questionnaire for Tic-Associated Sensations

**DOI:** 10.3389/fpsyt.2020.00592

**Published:** 2020-07-03

**Authors:** Natsumi Matsuda, Maiko Nonaka, Toshiaki Kono, Miyuki Fujio, Marina Nobuyoshi, Yukiko Kano

**Affiliations:** ^1^ Department of Child Neuropsychiatry, Graduate School of Medicine, The University of Tokyo, Tokyo, Japan; ^2^ Department of Developmental Psychology, Faculty of Human Studies, Shirayuri University, Tokyo, Japan; ^3^ Department of Child Psychiatry, The University of Tokyo Hospital, Tokyo, Japan; ^4^ Course of Clinical Psychology, Graduate School of Education, The University of Tokyo, Tokyo, Japan; ^5^ Department of Community Mental Health & Law, National Institute of Mental Health, National Center of Neurology and Psychiatry, Tokyo, Japan; ^6^ Department of Psychology, Faculty of Liberal Arts, Teikyo University, Tokyo, Japan

**Keywords:** Tourette syndrome, tics, suppression, premonitory urge, behavioral therapy

## Abstract

Awareness of premonitory urge in Tourette syndrome (TS) may facilitate tic suppression; however, previous studies have not supported this observation. We aimed to clarify the relationship between tic-associated sensation and tic suppression by identifying the subtypes of tic-associated sensations, including the Premonitory Urge for Tics Scale (PUTS). We developed a new questionnaire called “Rumination and Awareness Scale for tic-associated sensations” (RASTS) to assess the two additional aspects of tic-associated sensations: the intensity of somatosensory hyperawareness and the ability to identify signals of emerging tics. Sixty-two individuals with TS participated in the study (mean age = 19.2 ± 10.3 years). All participants completed the RASTS, PUTS, and Tic Suppression Scale. Of all participants, 41 were evaluated by the Yale Global Tic Severity Scale (YGTSS), while another group of 41 completed both the Leyton Obsessional Inventory-Child Version (LOI-CV) and the Tics Symptom Self-Report (TSSR). Factor analyses including nine items of the PUTS and the RASTS were conducted, and their relationships with patients’ tic suppression ability were examined. The results support using RASTS for the two supposed dimensions (rumination about sensation and premonitory awareness) for assessing the two different tic-associated sensations, and PUTS for three dimensions for assessing the two types of quality of premonitory urges and intensity of premonitory urges. Premonitory awareness correlated with tic suppression ability. Conversely, rumination about sensation, PUTS total score, and the three subscales of PUTS correlated with obsessive-compulsive symptoms. In summary, being aware of signals for emerging tics facilitated self-initiated tic suppression, while ruminative tic-associated sensations did not. This study provides new insights into behavioral therapy for tics by identifying two distinct aspects of tic-associated sensations that include premonitory urges.

## Introduction

Following Bliss, a physician with Tourette syndrome (TS), describing his own experience of sensory phenomena as “unfulfilled sensations that precede, accompany, and follow tics” in 1992 (1), many studies have revealed that premonitory urges are common phenomena in individuals with TS (1–5). Recently, premonitory urges have been examined in relation to tic suppressibility. Premonitory urges are often assessed by using the Premonitory Urge for Tics Scale (PUTS) (3). Using PUTS, three studies showed that premonitory urges did not correlate with tic suppressibility. Ganos et al. did not find a correlation between tic suppression ability and PUTS in 15 adults with TS (6), neither did Müller-Vahl et al., in 22 adults with TS (7). Conelea et al. examined predictors of tic suppressibility in 99 youth with tic disorders and did not find a significant correlation between tic suppressibility and PUTS (8). These three studies assessed tic suppressibility by comparing the tic frequency in “free tic” (no suppression) *vs*. “tic suppression” (instructed to maximally suppress their tics) conditions. In addition, Banaschewski and colleagues assessed the tic suppression ability of 254 children and adolescents with TS, and asked whether they felt a pre-sensation immediately before tics. Only 37% of patients with TS reported premonitory urges; however, 64% reported that they could suppress tics (9). Moreover, Woods et al. found no positive correlation between the nine-item PUTS total score and the self-reported tic suppressibility (PUTS item 10) in youth with TS, when they developed the PUTS scale (3), while dropping item 10 to calculate the total PUTS score. These studies indicate that premonitory urges are not necessary prerequisites for tic suppression.

However, awareness of premonitory urges seems to facilitate tic control in behavioral therapy. Awareness training for the premonitory urge is an essential technique in Habit Reversal Training (HRT). HRT is a core component of the Comprehensive Behavioral Intervention for Tics (CBIT), and CBIT has been proved effective for reducing tic symptoms in a large randomized control study in children and adolescents (10), and adults (11). Indeed, 18% of the 132 participants with TS reported that awareness of the premonitory urge helped them suppress their tics (2). Himle et al. evaluated the real-time subjective premonitory urge strength using the “urge thermometer” and investigated its relationship with tic suppression in five children and adolescents with TS (12). One out of five participants could not suppress her tic symptoms in the reinforced tic suppression conditions compared to the baseline conditions, while a relatively stable and low premonitory urge was observed during both base-line and tic suppression conditions. The other four participants suppressed their tics in the tic suppression conditions and reported a higher premonitory urge compared to the female participant during both conditions. In addition, Raines et al. found modest positive correlations with the nine-item PUTS total scores and self-reported tic suppressibility (PUTS item 10) in youth with TS (13). These studies imply that awareness of premonitory urge may facilitate tic suppression.

Inconsistent results may rise from a large variety of tic-associated sensations. As noted above, many studies evaluated premonitory urges by using the PUTS (3, 6–8, 13). The PUTS is a brief self-reported scale with good internal consistency, temporal stability, and concurrent validity in children and adolescents (3, 13), and adults (14), and with a good convergent validity in adults (15). Prior studies conducted a factor analysis of PUTS and indicated a unidimensional structure (3, 14), two structures (13), or three structures (15). Brandt et al. conducted a factor analysis, which included the 10 PUTS scores and an average real-time subjective premonitory urge intensity score during 5-min experiments (15), and showed three factors: one appearing to measure the urge intensity; one sensory quality of urges; and one subjective control. This suggests that PUTS might assess more than one dimension and it may be worthwhile exploring its different subscales. Raines found similar structures for PUTS and suggested that one factor represents the specific urge qualities while another represents the general urge experiences (13). Brandt et al. emphasized the need to investigate the underlying dimensions of premonitory urges in future studies (15).

Therefore, we aimed to explore the dimensions of tic-associated sensations and examine their relationship with tic suppression. First, we conducted a factor analysis of PUTS and explored the dimensions of the premonitory urge. Inconsistent findings of dimensions of premonitory urge have made it difficult to comprehend the underlying dimensions of tic-associated sensations. Second, we developed a new questionnaire to assess the other two aspects of tic-associated sensations, which PUTS does not assess, and examined the relationship with the tic suppression ability. PUTS of nine items includes six items for describing the different sensory qualities of urge, such as itchiness and tense of energy (item 1–6), and two items for describing how frequent the urges come with their tics (item 7 and 8), as well as the patients’ relief experienced following an executed tic (item 9) (3). Although PUTS is a commonly used scale for the tic-associated detail quality of sensory phenomena description, we also propose two additional important aspects of tic-associated sensations, including the somatosensory hyperawareness severity (rumination about sensation) and the ability to identify signals of emerging tics (premonitory awareness).

We focused not only on the pre-tic sensations but also on the ruminative tic-associated sensations, which may last following the tics. Kane, a graduate student with TS, noted that the term, “pre-tic” sensation is a misnomer as the TS feeling is acute and omnipresent (16). Kane also described the tic-associated sensations as “enduring somatosensory bombardment” and “somatosensory hyper attention.” As noted above, Bliss described his sensory phenomena as “unfulfilled sensations that precede, accompany, and follow tics” and did not focus only on the pre-tic sensation (1). However, following Leckman et al., using the term “premonitory urge” (2), many researchers have focused only on the pre-tic sensations (3–9) while underestimating the ruminative tic-associated sensations that may last even after the tics. The University of São Paulo Sensory Phenomena Scale (USP-SPS) evaluates sensory phenomena occurring before or during the performance of repetitive behaviors (17). Although USP-SPS includes broader sensory phenomena including the premonitory urge, the scale was first developed to assess the sensory phenomena in OCD and includes the sensory phenomena before compulsions (17). Therefore, we created a new scale to assess the somatosensory hyperawareness severity associated with tic symptoms, evaluated by how individuals with TS ruminate the tic-associated sensations (rumination about sensation). This ruminative sensation may be affected by obsessive-compulsive symptoms, as previous studies reported that obsessive-compulsive symptoms correlate with premonitory urges (3, 5, 18).

The second additional aspect of tic-associated sensation is the ability of signal identification for the emerging tics (premonitory awareness). Some individuals with TS have noted that they can tell when a tic will occur and suppress it before it emerges; however, they do not feel any specific sensation. Indeed, Banaschewske et al. showed that several participants could suppress tics without reporting the premonitory urge (9). During HRT, practitioners encouraged patients to identify signals for the emerging tics to initiate a competing response before the tic’s emergence (11). We assumed this signal of emerging tics to be related to the tic suppression ability.

Therefore, we created a new scale to assess the two aspects of tic-associated sensation (premonitory awareness and rumination about sensation). We also examined each trait concerning the tic suppression ability. We hypothesized that premonitory awareness correlates significantly with the tic suppression. In contrast, we hypothesized that PUTS is not related to the tic suppression ability, as previously reported (6–8), but correlates with the obsessive-compulsive symptoms, consistent with other reports (3, 5, 18). We hypothesize that rumination about sensation correlates with PUTS and obsessive-compulsive symptoms, and does not correlate with the tic suppression ability.

## Methods

### Participants

**Figure 1:**
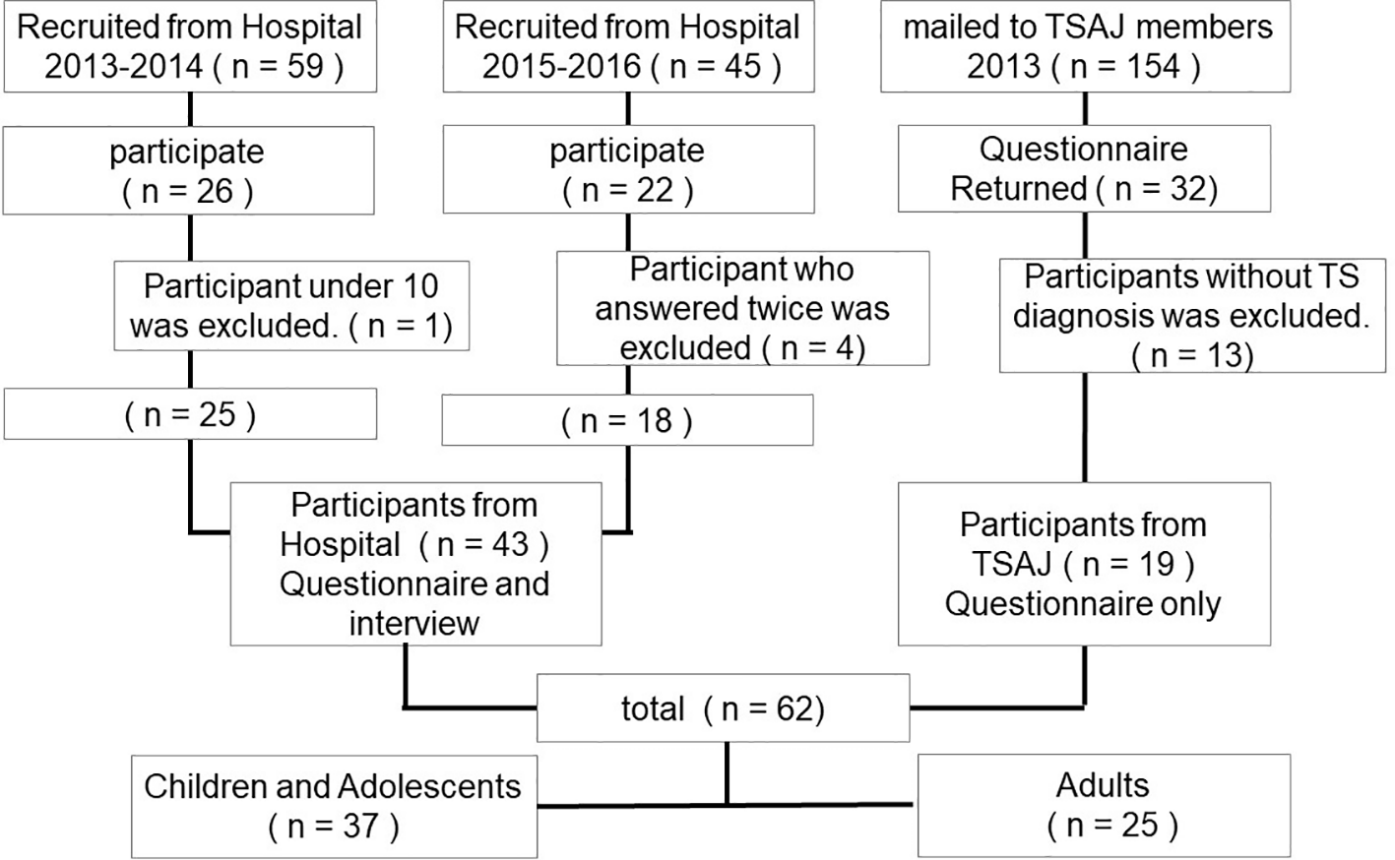
Participants flow-chart.

Participants were recruited from 2013 to 2016 in three steps (**Figure 1**). First, 59 patients with TS aged between 9 and 30 and their parents were recruited from the University of Tokyo Hospital from 2013 to 2014. Out of these, 26 patients with TS (44%) and the child participants’ parents participated following a full explanation of the study. Second, under the approval of the Tourette syndrome Association of Japan (TSAJ), 154 questionnaires were mailed to TSAJ members; consequently, 32 questionnaires (21%) returned from individuals with tic disorders and their parents. Third, 45 participants with TS aged between 13 and 53 were recruited from the University of Tokyo Hospital, from 2015 to 2016. Of these participants, 22 patients with TS (49%) participated following a full explanation of the study. Patients from the University of Tokyo Hospital had been diagnosed with TS according to the Diagnostic and Statistical Manual, Fourth Edition, Text Revision (DSM-IV-TR) criteria by a child psychiatrist (19). We excluded participants under the age of 10 years. For participants from TSAJ members, we excluded questionnaires that did not report a TS diagnosis. In addition, four clinic participants answered the questionnaire twice; consequently, we excluded the 2^nd^ questionnaire. Taken together, a total of 62 questionnaires were analyzed (43 clinic participants and 19 non-clinic participants; 50 men, 12 women, mean age = 19.2 years, *SD* = 10.3 years, range = 10–53 years). For the TSAJ participants and 22 of the clinic participants, their parents also answered a questionnaire, including the TSSR and diagnostic information (n = 41). We also evaluated YGTSS (20) for 41 participants recruited from the hospital. Regarding the clinic participants, written informed consent was obtained from the adults and parents of participating children, and assent was obtained from the children. For non-clinic participants, a response to the questionnaire that included a full explanation of the study was recognized as informed consent. The institutional ethical committee of the University of Tokyo Hospital approved the study protocol.

### Measures

The questionnaire consisted of RASTS, the Tic Suppression Scale, PUTS, and LOI-CV for participants with TS and TSSR, and diagnostic information for the parents of the participants with TS. YGTSS was evaluated for the participants from the hospital.

#### Rumination and Awareness Scale for Tic-Associated Sensations

This scale was designed to assess the two distinct aspects of the tic-associated sensation. We developed RASTS as follows: a set of questions were developed based on the clinical interviews with patients, consultation with experts, and a review of the relevant literature regarding the premonitory awareness and rumination about sensation. We referred to a negative rumination scale (21) in order to develop items evaluating somatosensory hyper-awareness severity. Participants answered 14 items (7 items for each; see **Supplementary Material**), and each item was scored from 0 to 5. To facilitate the participants in recalling their tic-associated sensations, they first completed the PUTS, in which detailed examples of description of tic-associated sensations were described.

#### The Tic Suppression Scale

We used a list of questions that addressed the three aspects of the tic suppression (see **Supplementary Material**) as follows: frequency (three items; Cronbach’s *α* = 0.87), suppression ability (six items; Cronbach’s *α* = 0.92), and subjective discomfort (three items; Cronbach’s α = 0.87). Participants responded using a 4-point Likert-type scale ranging from 0 to 3. These items were also used in our previous study (22), except for the tic suppression ability which was improved by adding four extra items that evaluated the distinct tic suppression ability aspects in this study. The validity and usefulness of the Japanese version of the Tic Suppression Scale was tested by demonstrating a negative correlation with a motor tic interference scale of the YGTSS and a positive correlation with overall satisfaction with tic control (23).

#### Premonitory Urge for Tics Scale

The PUTS is a nine-item self-reported scale that measures the sensations before the emergence of tics, and each item is scored from 1 to 4 (3). The total score (range: 9–36) is obtained by summing all of the nine items. The Japanese version of the scale was designed using rigorous methods, including translation and back translation, and with sufficient internal and concurrent validity (18).

#### The Yale Global Tic Symptoms Scale

The presence and severity of tics were evaluated in the recruited clinic participants using the Japanese version of the Yale Global Tic Symptoms Scale (YGTSS) (20). This version has previously been proven valid and reliable (24). On this scale, the motor and vocal tics were evaluated separately (0–25) on 5 ordinal scales, and a total tic symptom score was obtained by summing the individual scores (0–50). The current impairment due to tics was additionally assessed (0–50). The global severity score was determined (0–100) as the sum of the total tics score and the impairment score.

#### The Tic Symptom Self Report

The Tic Symptom Self Report (TSSR) is a 40-item self- or parent-rated scale measuring the tic severity, with items scored from 0 to 3. We adopted the parent-rated version (25). Participants rated the frequency and intensity of the list of symptoms (20 for motor tics and 20 for vocal tics); then, a summed tic severity score was calculated (0 to 120). The validity and usefulness of the Japanese version of the TSSR was tested by demonstrating a high correlation with the YGTSS (26). We used the TSSR to only compare the tic symptoms between the clinic patients and the TSAJ members.

#### The Leyton Obsessional Inventory Child Version

The Leyton Obsessional Inventory Child Version (LOI-CV) is a self-reported 20-item scale measuring the severity of obsessive-compulsive symptoms (27). In this inventory, participants report whether they experience any of the 20 symptoms that have been previously reported, as well as how these symptoms interfere with their daily activities. Both the validity and reliability of the Japanese version of the LOI-CV have been demonstrated (28).

Other characteristics, including the participants’ age, sex, diagnoses of TS, attention deficit hyperactive disorder (ADHD), and obsessive-compulsive disorder (OCD) was obtained from the parents’ reported questionnaire for the participants from TSAJ. For the participants recruited in the hospital, comorbid OCD and ADHD were diagnosed based on the DSM-IV-TR criteria, as in the case of TS (19).

### Statistical Analysis

Statistical analyses were performed using the SPSS software version 25.0. Differences were considered statistically significant if *p <*0.05. Exploratory factor analysis for the RASTS was conducted. In addition, we conducted an exploratory factor analysis to explore the PUTS dimensions. Spearman’s rank correlation coefficients were calculated to examine the relationships between each clinical characteristic, as some scales were not normally distributed, including the premonitory awareness, rumination about sensation, tic suppression frequency, and subjective discomfort according to the Shapiro-Wilk’s normality test (29). We applied Bonferroni corrections for multiple comparisons. To examine the relationship between the three scales of tic-associated sensation (PUTS, premonitory awareness, and rumination about sensation) and tic suppression ability, the significance level was divided by three (*p <*0.017). To examine the relationship between the three tic-associated sensation scales and the obsessive-compulsive symptoms, the significance level was further divided by three (*p <*0.017). Missing values were replaced by estimated values calculated by regression analysis using other items on the scale. We conducted a factor analysis of PUTS and Spearman’s rank correlation by the age group (children and adolescents for age ≤17 and adult for age ≥18) to find the possible effect of age. To explore the effect of comorbid disorders, we conducted a correlation analysis of tic suppression and tic-associated sensations by the two groups (participants without comorbid disorders or participants with ADHD or OCD).

## Results

There was no significant difference in the tic severity (evaluated by the TSSR) between the clinical participants (mean = 13.4, *SD* = 9.3) and TSAJ members (mean = 18.8, *SD* = 12.1, *t* (39) = –1.64, *p* = 0.11). Therefore, we did not separate the clinical participants and TSAJ members in the following analysis. **Table 1** describes the participants’ demographic and clinical data for the total group, the children and adolescent group, and the adult group. There were no significant differences in the PUTS, two RASTS scales, LOI-CV, total tic symptom score, and the global severity score of YGTSS between the children and adolescent participants and the adult participants, when using the t-test. Impairment scores of YGTSS and the ability to suppress tics were higher in the adult group compared to the children and adolescent group (*t* (39) = –2.4, *p* = 0.02, *t* (60) = –2.8, *p* = 0.007, respectively). Gender differences and comorbid disorders (ADHD and OCD) were compared between the two groups using the chi-square test, which indicated no significant differences (*p* = 0.24–0.51).

**Table 1:** Demographic and clinical data for the participants.

		Total			Children and adolescents			Adults		
		Mean	SD	Range	Mean	SD	Range	Mean	SD	Range
Age		19.2	(10.3)	10–53	12.9	(2.4)	10–17	28.5	(10.5)	18–53
PUTS		21.9	(6.9)	9–35	21.2	(6.9)	9–34	22.9	(6.9)	10–35
Rumination and Awareness Scale for tic-related sensation										
	Rumination about sensation	2.3	(1.6)	0–5	2.3	(1.6)	0–5	2.2	(1.6)	0–5
	Premonitory awareness	3.6	(1.3)	0.6–5	3.4	(1.4)	0.6–5	3.8	(1.3)	1–5
Tic Suppression Scale										
	Frequency to suppress	1.6	(0.9)	0–3	1.4	(0.9)	0–3	1.9	(0.8)	0.3–3
	Ability to suppress	1.5	(0.8)	0–3	1.3	(0.8)	0–2.8	1.8	(0.7)	0.4–3
	Discomfort from suppression	1.9	(0.9)	0–3	1.8	(0.9)	0–3	1.9	(0.9)	0–3
YGTSS										
	Tic symptoms	21.1	(7.7)	5–37	19.4	(7.2)	5–30	23.4	(7.8)	11–37
	Impairments	19.0	(10.7)	0–40	15.7	(9.0)	0–30	23.3	(11.4)	0–40
LOI-CV		22.7	(13.5)	2–53	22.2	(13.8)	4–53	24.1	(13.1)	2–43
TSSR		15.9	(10.9)	0–40	16.2	(11.0)	0–40	14.9	(11.2)	2–30
Gender distribution (male/female)		50/12			31/6			19/6		
Comorbidity										
	OCD	15	24%		7	19%		8	32%	
	ADHD	17	27%		9	24%		8	32%	

n = 62 for total (n = 37 for the children and adolescents, n = 25 for adults), n = 41 for YGTSS, LOI-CV, TSSR.

### Factor Analysis for Rumination and Awareness Scale for Tic-Associated Sensations

An exploratory factor analysis (maximum likelihood method: MLM) with an oblique rotation method was conducted to determine the factor structure of the 14 items of RASTS. Consideration of the criteria of the absolute value of the eigenvalues, visual screening test, and theoretical interpretability of the factors suggested a two-factor solution. One primary factor accounted for 34.4% of the variance in the data and another factor accounted for an additional 22.3% of the variance. Therefore, we conducted an MLM factor analysis in which we specified a two-factor solution. Items were removed according to the following criteria: a) items should exceed 0.4 on the corresponding factor and b) items should not exceed 0.25 on another factor. Consequently, three items were removed from the RASTS as they did not meet these criteria (items 8, 9, and 11). Another MLM exploratory factor analysis with oblique rotation was conducted with the remaining 11 items, where 2 factors accounted for 39.2 and 25.6% of the variance, respectively, and 64.8% of the variance cumulatively. The first factor with six items was labeled “rumination about sensation” and the second factor with five items was labeled “premonitory awareness” (**Table 2**). Both measures indicated adequate internal consistency, with Cronbach’s *α* = 0.91 for the rumination about sensation (*α* = 0.91 both in the young and adult sample) and Cronbach’s *α* = 0.79 for premonitory awareness (*α* = 0.80 in the young sample and *α* = 0.79 in the adult sample). The correlation between the two factors was measured at −0.03 (*p* = 0.83).

**Table 2:** Factor analysis of Rumination and Awareness Scale for tic-associated sensations (RASTS).

Item	Mean (SD)		Factor 1	Factor 2
Rumination about sensation				
12. Sometimes, I just keep feeling bothered by the sensation for over 30 min without a break.	1.69	(1.98)	0.88	−0.08
14. Often, I just can’t stop focusing on a sensation.	2.06	(2.00)	0.87	0.05
10. Sometimes, I feel bothered by a sensation all day long.	1.50	(1.85)	0.82	−0.06
4. Once the sensation has emerged, I will just keep being bothered by it.	2.71	(1.95)	0.75	−0.01
6. Sometimes, I focus on the sensation for dozens of minutes.	2.40	(1.91)	0.75	−0.02
1. After the sensation emerges, I’m likely to be bothered by it repeatedly, again and again.	3.18	(1.60)	0.67	0.10
Mean	2.26	(1.57)	Range: 0–5	
Premonitory awareness				
13. I notice tics before they appear, tics do not happen automatically.	3.52	(1.85)	0.08	0.94
5. Before I experience a tic, I know what kind of tic it will be.	3.74	(1.74)	0.11	0.67
2. When the tic is about to appear, I can often notice it.	4.03	(1.41)	0.14	0.64
7*. I often experience a tic without noticing it.	3.39	(1.99)	−0.21	0.61
3*. In some cases, I don’t notice that the tic has appeared.	3.27	(1.92)	−0.21	0.49
Mean	3.59	(1.33)	Range: 0.6–5	

The refined 11-item Rumination and Awareness Scale for tic-associated sensations (RASTS) is presented here, with three items were removed; n = 48; factors were extracted using maximum likelihood method (MLM) analysis with Oblique rotation. Items are presented with their loadings onto their assigned factor.

*Items for reverse scoring.

### Factor Analysis for Premonitory Urge for Tics Scale

Cronbach’s α across nine items of the PUTS were acceptable in the total sample (α = 0.85), young sample (α = 0.85), and adult sample (α = 0.87). An exploratory factor analysis (maximum likelihood method) with an oblique rotation method was conducted to determine the factor structure of the nine items of PUTS, indicating three factors. One factor accounted for 36.4% of the variance, two factors accounted for 56.4% of the variance, and three factors accounted for 65.2% of the variance. For total participants, items 1, 2, and 3 loaded on the first factor, items 1, 7, and 8 loaded on the second factor, and items 4, 5, and 9 loaded on the third factor. The same exploratory factor analysis was conducted for the young participant group and the adult participant group, resulting in a similar result (**Table 3**). For the young sample, items 1, 2, and 3 loaded on the first factor, items 1, 7, and 8 loaded on the second factor, and items 4, 5, and 9 loaded on the third factor. For the adult group, items 1, 2, and 3 loaded on the first factor, items 7 and 8 loaded on the second factor, and items 1, 4, 5, and 9 loaded on the third factor.

**Table 3:** Factor analysis of Premonitory Urge for Tics Scale (PUTS).

	Total			Children and adolescent group			Adult group		
	F1	F2	F3	F1	F2	F3	F1	F2	F3
Item 1 Right before I do a tic, I feel like my insides are itchy.	**0.48**	**0.40**	−0.03	**0.44**	**0.54**	−0.12	**0.43**	−0.02	**0.40**
Item 2 Right before I do a tic, I feel pressure inside my brain or body.	**0.79**	0.08	−0.11	**0.74**	0.18	−0.07	**0.79**	−0.09	−0.05
Item 3 Right before I do a tic, I feel “wound up” or tense inside.	**1.03**	−0.18	0.03	**1.07**	−0.20	0.07	**1.08**	0.06	−0.22
Item4 Right before I do a tic, I feel like something is not “just right.”	−0.02	0.05	**0.82**	−0.07	0.11	**0.77**	0.09	−0.05	**0.89**
Item 5 Right before I do a tic, I feel like something isn’t complete.	0.03	0.09	**0.92**	0.06	0.09	**0.93**	0.08	0.19	**0.78**
Item 6 Right before I do a tic, I feel like there is energy in my body that needs to get out.	0.16	0.29	0.15	0.18	0.30	0.16	0.07	0.23	0.30
Item 7 I have these feelings almost all the time before I do a tic.	0.03	**0.86**	0.06	−0.02	**1.00**	0.02	0.01	**1.04**	−0.07
Item 8 These feelings happen for every tic I have.	−0.10	**0.96**	−0.10	−0.08	**0.76**	0.04	−0.08	**0.76**	0.12
Item 9 After I do the tic, the itchiness, energy, pressure, tense feelings, or feelings that something isn’t “just right” or complete go away, at least for a little while.	−0.08	−0.11	**0.55**	−0.01	−0.10	**0.61**	−0.27	−0.01	**0.52**

Total, n = 62; for children and adolescents, n = 37; for adult, n = 25. The largest loading factors are written in bold font.

### Correlation Between Premonitory Urge and Rumination and Awareness Scale for Tic-Associated Sensations

The rumination about sensation and the three subscales of the PUTS score and total PUTS score significantly correlated with each other, while there were no significant correlations between the premonitory awareness and other tic-associated sensation scales (rumination about sensation, three subscales of PUTS score, or PUTS total scores) (**Table 4**). The rumination about sensation and PUTS scores were significantly correlated with the LOI-CV score (*ρ* = .50, *p* <.001; *ρ* = .52, *p* <.001, respectively), while the premonitory awareness score did not correlate with the LOI-CV score (*ρ* = .11, *p* = .50). In addition, the rumination about sensation and PUTS scores were significantly correlated with YGTSS total tic symptom score (*ρ* = .35, *p* = .02; *ρ* = .32, *p* = .04, respectively), while the premonitory awareness score was not correlated with the YGTSS total tic symptom score (*ρ* = −.21, *p* = .18). These correlation analyses were conducted in the adult and the young group separately, and they exhibited similar correlation patterns.

**Table 4:** Spearman’s Correlation between Premonitory Urge for Tics Scale (PUTS) and Rumination and Awareness Scale for tic-associated sensations (RASTS).

	Rumination about sensation	PUTS	PUTS	PUTS	PUTS	LOI-CV	Age	YGTSS	YGTSS
		Total	Physical	Intensity	Just right			Symptoms	Total severity
Premonitory awareness	−.03	.00	−.09	.02	.10	.11	.28*	−.21	−.19
Rumination about sensation		.72***	.49***	.64***	.59***	.50***	−.11	.35*	.29
PUTS total			.73***	.84***	.82***	.52***	.08	.32*	.38*
PUTS physical				.48***	.33**	.48**	.07	.17	.25
PUTS intensity					.62***	.37*	.03	.25	.34*
PUTS just right						.41**	.14	.45**	.41**
LOI-CV							.17	.28	.18
Age								.10	.21

For LOI-CV and YGTSS, n = 41; for items expect for LOI-CV and YGTSS, n = 62.

PUTS, Premonitory Urge for Tics Scale; LOI-CV, Leyton Obsessional Inventory Child Version; RASTS, Rumination and Awareness Scale for tic-associated sensations.

*p < 0.05, **p < 0.01, ***p < 0.001.

### Correlation Between Tic Suppression and Premonitory Urge and Rumination and Awareness Scale for Tic-Associated Sensations

**Figure 2:**
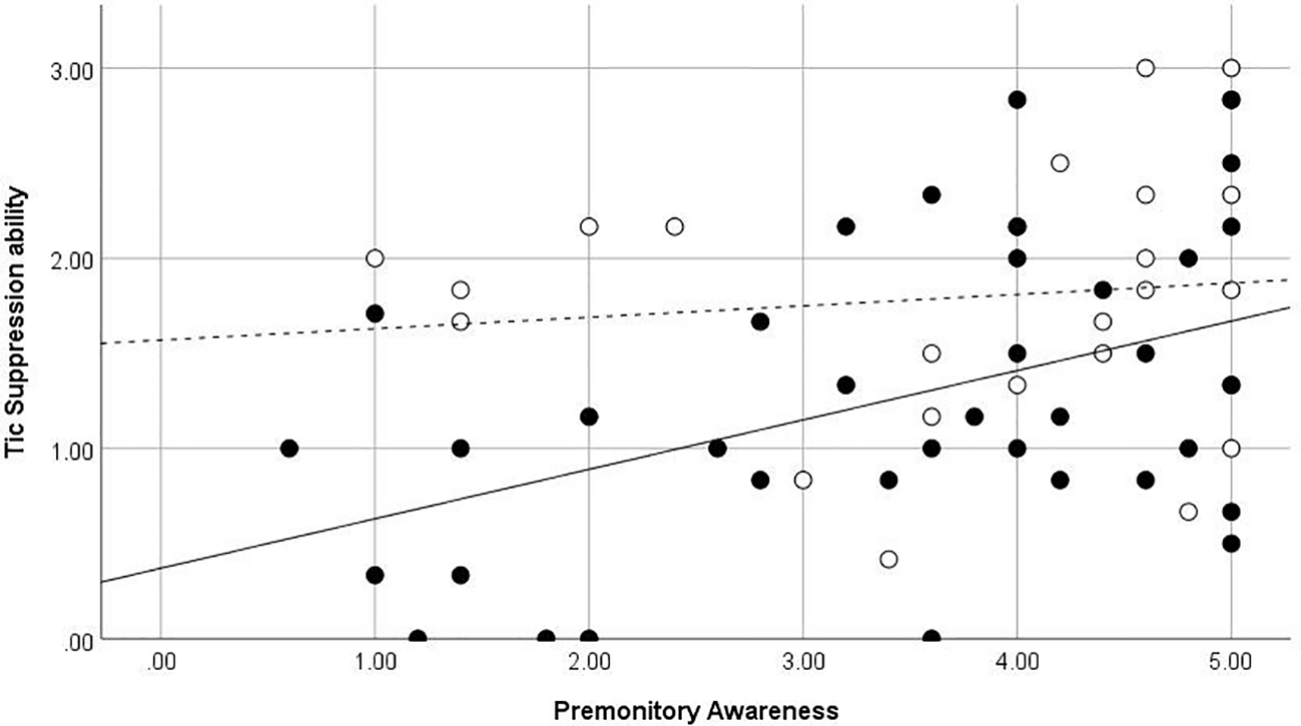
Premonitory Awareness and Tic Suppression ability. ●, Children and adolescents, straight line (—) is for regression line in children and adolescent, dot line (- - -) is for regression line in adult participants ○, Adults. Regression line in adult participants.

The premonitory awareness score was significantly correlated with the tic suppression ability (*ρ* = .34, *p* = 0.008). The rumination about sensation and PUTS scores did not correlate with the tic suppression ability (*ρ* = –.25, *p* = .052; *ρ* = –.22, *p* = .09, respectively), consistent with our hypothesis (**Table 5**). In the young group, the premonitory awareness score was significantly correlated with the tic suppression ability (*ρ* = .39, *p* = .019). However, the premonitory awareness score was not significantly correlated with the tic suppression ability in the adult group (*ρ* = .19, *p* = .38). **Figure 2** shows the scatter plot of the relationship between the premonitory awareness and rumination about sensation and the tic suppression ability for the adult and young groups. In the groups without OCD or ADHD (*n* = 37), rumination about sensation and PUTS scores significantly correlated negatively with the tic suppression ability (*ρ* = –.41, *p* = .012; *ρ* = –.40, *p* = .015, respectively), and the premonitory awareness correlated with tic suppression ability (*ρ* = .36, *p* = .028) although p score was slightly higher than 0.017 (modified significant level). In the group with ADHD or OCD diagnosis (*n* = 25), the PUTS, premonitory awareness, and rumination about sensation did not correlate with the tic suppression ability (*p* = .17~.99).

**Table 5:** Spearman’s correlation between tic-associated sensations and tic suppressibility.

		Tic suppression scale			
		Frequency	Suppression ability	Subjective discomfort	Satisfaction with tic control
Total	Premonitory awareness	.12	.34**	−.21	.04
	Rumination about sensation	.33**	−.25	.56***	−.38**
	PUTS total	.30*	−.22	.61***	−.36**
Children and adolescents	Premonitory awareness	.06	.39*	−.25	.09
	Rumination about sensation	.25	−.31	.54***	−.53***
	PUTS total	.28	−.30	.55***	−.45**
Adult	Premonitory awareness	.17	.19	−.23	.00
	Rumination about sensation	.40	−.09	.63***	−.14
	PUTS total	.27	−.12	.66***	−.23

Total, n = 62; for children and adolescent group, n = 37; for adult group, n = 25.

PUTS, Premonitory Urge for Tics Scale, * p < 0.05, **p < 0.01, ***p < 0.001.

## Discussion

### Premonitory Urge in Japanese Participants

Premonitory urge in Tourette syndrome has not been fully investigated in Asia. The PUTS score of our result was 21.9 out of 36 (21.2 for children and adolescents and 22.9 for adults). This score is similar to the initial report (18.5 for children and adolescents) (3) and the following reports [19.6 for children and adolescents, (13), 21.5 for adults, (14)], which are reported in the USA. Moreover, this score is similar to a previous Japanese study (18.6 for children and adolescents and adults) (18). Consistent with previous reports (3, 14, 18), our study showed a moderately positive correlation between the PUTS scores and the YGTSS total tic symptom score and obsessive-compulsive symptoms. These results indicate that Japanese individuals with TS feel similar premonitory urges compared to individuals with TS in Western cultures.

### Dimensions of Tic-Associated Sensations

Factor analysis of the 9 item PUTS revealed three factors, which is consistent with a previous study that conducted a factor analysis of the 10 item PUTS. Items loading on the first factor (items 1, 2, and 3) assess whether participants with TS feel “itchy,” “pressure,” or “tense” before a tic, potentially reflecting the physical quality of the premonitory sensations. Items loading on the second factor (items 7 and 8) assess the extent to which the participants had these “feelings almost all the time” before tics and “for every tic,” which are related to the overall intensity of the premonitory urges. Item 1 also loaded on the first factor in the total group and the young group. Items loading on the third factor (items 4, 5, and 9) assess whether participants feel “not just right” or “something isn’t complete” before a tic, or how much relief of sensation comes after the tic occurs. Our results showed that the first and second factors reflect the quality of the premonitory urge and the intensity of premonitory urges, respectively, similar to the previous factor analysis of PUTS (13, 15). In contrast to a previous study (15), our third factor also includes the just right sensation (items 4 and 5) in addition to the relief from the premonitory urge following the tic (item 9). This result may partly come from a different number of items that were used for the factor analysis. We used nine items of PUTS, consistent with the original version. Brandt et al. used all 10 items of PUTS and their original real-time urge intensity score (15) whereas Raines et al. used 8 items of PUTS while excluding item 9 (13).

Correlation analysis between PUTS and our newly created tic-associated sensations scale suggested that the three PUTS subscales and ruminative tendency toward tic-associated sensations correlated with each other; however, pre-tic awareness was not related to any other tic-associated sensations. Upon conducting an exploratory analysis, two items including the “sensation”, were excluded from the premonitory awareness factor (item 9, “I experience more tics with a prior sensation than I do tics without warning,” and item 11, “Often, I feel some kind of sensation before the tic appears.”), and items not including the sensations but referring their awareness before the tics were retained. It indicates that individuals with TS do not have to feel any concrete sensations before tics to tell when their tic will occur.

### Relation Between Tic-Associated Sensations and Tic Suppression Ability

Consistent with previous studies (6–8), PUTS scores were not correlated with tic suppression ability. PUTS scores were significantly correlated with obsessive-compulsive symptoms, consistent with previous reports (3, 5, 18). Rumination about tic-associated sensation was not correlated with the tic suppression ability but was significantly correlated with obsessive-compulsive symptoms. In the group of participants without ADHD nor OCD, PUTS and rumination about sensation was negatively correlated with the tic suppression ability. Therefore, we concluded that aversive tic-associated somatosensory experiences do not facilitate tic suppression or even make the tic suppression more difficult. In contrast, the ability to tell when tic will occur before participants experience tics was correlated positively with the tic suppression ability, and this awareness may help tic suppression.

When the analysis was conducted by the age group, the correlation between the premonitory awareness and tic suppression was significant only in the group with the TS children. An additional different relationship between the urges and tic suppression was observed in the different habituation of the premonitory urge in CBIT practice between the children and adults with TS (30). Houghton et al. examined the habituation of premonitory urge for the CBIT responders and the CBIT non-responders with chronic tic disorders and revealed that adult CBIT responders exhibited some degree of premonitory urge severity reductions; however, child CBIT responders failed to show any significant reduction in the premonitory urges (30). The authors partially explain these differences by hypothesizing on the development and maintenance of the premonitory urges. First, children with tics may fail to recognize the urges or experience them as non-aversive; however, as tics continue to occur and increase in severity, they result in aversive consequences i.e., their urges being associated with tics acquires aversive valence (3, 30, 31). We created a premonitory awareness scale to evaluate the more neutral aspects of tic-associated sensation. Indeed, premonitory awareness did not correlate with the tic severity and obsessive-compulsive symptoms, and the subjective discomfort from tic suppression. However, it may be possible that premonitory awareness gains aversive valence as participants get older, affecting the tic-suppression ability in older participants.

### Limitation and Future Directions

Some limitations should be noted. First, the participants were recruited through only one specialty clinic for TS and related disorders or members of TSAJ, which may have led to biased and small sample size. Further, we included both pediatric and adult patients from the clinic. Although we divided all participants by age group and conducted the analyses by age group, the number of participants in each group was small. As Banaschewski et al. demonstrated, awareness of premonitory sensory phenomena develops with advancing age (9). Further studies with an increased number of participants for both children and adolescents are required to clarify the effect of developmental steps on the relationship between the tic-associated sensations and tic suppression. Second, we evaluated the tic suppression ability and premonitory awareness by using self-reported measures. We asked the participants to report RASTS and fill in the tic suppression questionnaire only once. Further studies are required to ensure test-retest reliability and concurrent validity to use the experimental evaluation of both the tic suppression ability and the ability to realize signals of emerging tics. Third, we recruited adult participants with TS mainly from the hospital, and this may lead to a possible confounding factor of the effect of age on the relationship between the tic-associated sensation and tic suppression. A higher percentage of ADHD in adults compared to children and adolescents may be caused due to this recruitment method. Fourth, we used the LOI-CV to evaluate the obsessive-compulsive symptoms. Although the relationship between the obsessive-compulsive symptoms and PUTS scores were consistent with those reported in previous studies, the results concerning obsessive-compulsive symptoms should be interpreted with caution. Further studies using Y-BOCS (32) to evaluate obsessive-compulsive symptoms are required.

In conclusion, to the best of our knowledge, this is the first study to examine the relationship between the tic suppression ability and the premonitory awareness and rumination about sensation. Premonitory awareness was not correlated with any other tic-associated sensations; conversely, PUTS and rumination about sensation correlated with each other. Our results indicate that the severity and frequency of tic-associated sensations (evaluated by PUTS and rumination about sensation) do not facilitate the tic suppression. In contrast, the awareness of signals for emerging tics facilitates the self-initiated tic suppression. This study provides additional insights into the mechanisms underlying the effectiveness of behavior therapy by clarifying two distinct aspects of tic-related somatosensory sensation that involve premonitory urges; repeated somatosensory phenomenon that is bothersome to individuals with TS and signals for emerging tics that may facilitate tic suppression in HRT. Future studies are required to examine these two distinct aspects of tic-associated sensations in the context of behavioral therapy.

## Data Availability Statement

The datasets generated for this study are available on request to the corresponding author.

## Ethics Statement

The institutional ethical committee of the University of Tokyo Hospital approved the study protocol. Written informed consent was obtained from the adults and parents of participating children, and assent was obtained from the children for clinic participants. For non-clinic participants, a response to the questionnaire that included a full explanation of the study was recognized as informed consent.

## Author Contributions

NM: conceptualization/design, data acquisition, analysis, data interpretation, draft the manuscript, MaiN, MF, and YK: data acquisition, critical revision of the manuscript, TK and MarN: critical revision of the manuscript. All authors contributed to the article and approved the submitted version.

## Funding 

This study was supported by JSPS KAKENHI Grant Number JP17J40054, JP18K13317.

## Conflict of Interest

The authors declare that the research was conducted in the absence of any commercial or financial relationships that could be construed as a potential conflict of interest.

## References

[1] Bliss J Cohen DJ Freedman DX Sensory experiences of Gilles-de-la-Tourette Syndrome. Arch Gen Psychiatry (1993) 37:1343–7. 10.1001/archpsyc.1980.01780250029002 6934713

[2] Leckman JF Walker DE Cohen DJ Premonitory urges in Tourettes Syndrome. Am J Psychiatry (1993) 150:98–102. 10.1176/ajp.150.1.98 8417589

[3] Woods DW Piacentini J Himle MB Chang S Premonitory urge for tics scale (PUTS): Initial psychometric results and examination of the premonitory urge phenomenon in youths with tic disorders. J Dev Behav Pediatr (2005) 26:397–403. 10.1097/00004703-200512000-00001 16344654

[4] Kwak C Dat Vuong K Jankovic J Premonitory sensory phenomenon in Tourette’s syndrome. Mov Disord (2003) 18:1530–3. 10.1002/mds.10618. 14673893

[5] Crossley E Cavanna AE Sensory phenomena: clinical correlates and impact on quality of life in adult patients with Tourette syndrome. Psychiatry Res (2013) 209:705–10. 10.1016/j.psychres.2013.04.019. 23684051

[6] Ganos C Kahl U Schunke O Kuhn S Haggard P Gerloff C Are premonitory urges a prerequisite of tic inhibition in Gilles de la Tourette syndrome? J Neurol Neurosurg Psychiatry (2012) 83:975–8. 10.1136/jnnp-2012-303033. 22842713

[7] Muller-Vahl KR Riemann L Bokemeyer S Tourette patients’ misbelief of a tic rebound is due to overall difficulties in reliable tic rating. J Psychosom. Res (2014) 76:472–6. 10.1016/j.jpsychores.2014.03.003 24840142

[8] Conelea CA Wellen B Woods DW Greene DJ Black KJ Specht M Patterns and predictors of tic suppressibility in youth with tic disorders. Front Psychiatry (2018) 9:188. 10.3389/fpsyt.2018.00188. 29875706 PMC5974106

[9] Banaschewski T Woerner W Rothenberger A Premonitory sensory phenomena and suppressibility of tics in Tourette syndrome: developmental aspects in children and adolescents. Dev Med Child Neurol (2003) 45:700–3. 10.1017/s0012162203001294. 14515942

[10] Piacentini J Woods DW Scahill L Wilhelm S Peterson AL Chang S Behavior therapy for children with tourette disorder a randomized controlled trial. JAMA (2010) 303:1929–37. 10.1001/jama.2010.607 PMC2993317 20483969

[11] Wilhelm S Peterson AL Piacentini J Woods DW Deckersbach T Sukhodolsky DG Randomized trial of behavior therapy for adults with tourette syndrome. Arch Gen Psychiatry (2012) 69:795–803. 10.1001/archgenpsychiatry.2011.1528. 22868933 PMC3772729

[12] Himle MB Woods DW Conelea CA Bauer CC Rice KA Investigating the effects of tic suppression on premonitory urge ratings in children and adolescents with Tourette’s syndrome. Behav Res Ther (2007) 45:2964–76. 10.1016/j.brat.2007.08.007 17854764

[13] Raines JM Edwards KR Sherman MF Higginson CI Winnick JB Navin K Premonitory Urge for Tics Scale (PUTS): replication and extension of psychometric properties in youth with chronic tic disorders (CTDs). J Neural Transm (2018) 125:727–34. 10.1007/s00702-017-1818-4. 29185077

[14] Reese HE Scahill L Peterson AL Crowe K Woods DW Piacentini J The premonitory urge to tic: measurement, characteristics, and correlates in older adolescents and adults. Behav Ther (2014) 45:177–86. 10.1016/j.beth.2013.09.002. PMC4445415 24491193

[15] Brandt VC Beck C Sajin V Anders S Munchau A Convergent Validity of the PUTS. Front Psychiatry (2016) 7:51. 10.3389/fpsyt.2016.00051. 27092085 PMC4823310

[16] Kane MJ Premonitory urges as attentional tics in Tourettes-Syndrome. J Am Acad Child Psy (1994) 33:805–8. 10.1097/00004583-199407000-00005. 8083137

[17] Rosario MC Prado HS Borcato S Diniz JB Shavitt RG Hounie AG Validation of the University of São Paulo Sensory Phenomena Scale: initial psychometric properties. CNS Spectr (2009) 14:315–23. 10.1017/s1092852900020319. 19668122

[18] Kano Y Matsuda N Nonaka M Fujio M Kuwabara H Kono T Sensory phenomena related to tics, obsessive-compulsive symptoms, and global functioning in Tourette syndrome. Compr Psychiatry (2015) 62:141–6. 10.1016/j.comppsych.2015.07.006. 26343478

[19] American Psychiatric Association Diagnostic and statistical manual of mental disorders. 4th ed Washington, DC: American Psychiatric Publishing (2000).

[20] Leckman JF Riddle MA Hardin MT Ort SI Swartz KL Stevenson J The Yale Global Tic Severity Scale: initial testing of a clinician-rated scale of tic severity. J Am Acad Child Psy (1989) 28:566–73. 10.1097/00004583-198907000-00015. 2768151

[21] Ito T Agari I Development of the negative rumination scale and its relationship with depression. Japanese J Couns Sci (2001) 34:31–42. (in Japanese).

[22] Matsuda N Kono T Nonaka M Fujio M Kano Y Self-initiated coping with Tourette’s syndrome: Effect of tic suppression on QOL. Brain Dev (2016) 38:233–41. 10.1016/j.braindev.2015.08.006 26360257

[23] Matsuda N Nonaka M Kono T Fujio M Kano Y Tic symptoms and tic suppressiblity in tourette’s syndrome. 58th Japanese Soc Child Adolesc Psychiatry (2017) 262. (in Japanese).

[24] Inoko K Nishizono-Maher A Tani S Kano Y Kishimoto J Hayakawa N Reliability and validity of a Japanese version of the Yale Global Tic Severity Scale: A preliminary study. Jpn J Child Adolesc Psychiatr (2006) 47(Supplement):38–48.

[25] Scahill L Leckman JF Schultz RT Katsovich L Peterson BS A placebo-controlled trial of risperidone in Tourette syndrome. Neurology (2003) 60:1130–5. 10.1212/01.wnl.0000055434.39968.67. 12682319

[26] Nonaka M Matsuda N Kono T Fujio M Kano Y Utilization of Tic Symptom Self Report; Testing validity and utilization for cognitive behavior therapy. 54th Japanese Soc Child Adolesc Psychiatry (2013) 428. (in Japanese).

[27] Berg CZ Whitaker A Davies M Flament MF Rapoport JL The survey form of the Leyton Obsessional Inventory-Child Version: norms from an epidemiological study. J Am Acad Child Psy (1988) 27:759–63. 10.1097/00004583-198811000-00017 3198565

[28] Hayashi Y Yoshihashi Y Okada R Tani I Onishi M Nakajima S Investigation on obsessive-compulsive tendencies of japanese children and adolescents: Using a Japanese version of the Leyton Obsessional Inventory-Child Version (LOI-CV-J). Japanese J Child Adolesc Psychiatry (2012) 53(1):1–10.

[29] Shapiro SS Wilk MB An analysis of variance test for normality (complete samples). Biometrika (1965) 52:591–611. 10.2307/2333709

[30] Houghton DC Capriotti MR Scahill LD Wilhelm S Peterson AL Walkup JT Investigating habituation to premonitory urges in behavior therapy for tic disorders. Behav Ther (2017) 48:834–46. 10.1016/j.beth.2017.08.004 PMC5679290 29029679

[31] Capriotti MR Espil FM Conelea CA Woods DW Environmental factors as potential determinants of premonitory urge severity in youth with Tourette syndrome. J Obsess-Compuls Rel (2013) 2:37–42. 10.1016/j.jocrd.2012.10.004

[32] Goodman WK Price LH Rasmussen SA Mazure C Fleischmann RL Hill CL The Yale-Brown Obsessive Compulsive Scale. 1. Development, Use, and realiability. Arch Gen Psychiatry (1989) 46:1006–11. 10.1001/archpsyc.1989.01810110048007 2684084

