# Supplementary material for: Premonitory Awareness Facilitates Tic Suppression: Subscales of the Premonitory Urge for Tics Scale and a New Self-Report Questionnaire for Tic-Associated Sensations
Source: Front Psychiatry. 2020 Jul 3;11:592. doi: 10.3389/fpsyt.2020.00592 (PMC7350852; doi:10.3389/fpsyt.2020.00592)
Supplement: Supplementary file 1 [file Presentation_1.pdf]

"Rumination and Awareness Scale for Tic-associated sensations" (RASTS)

Do you experience any sensation prior to tics, such as itchiness, increased energy or pressure, feeling wound up, tense, not just right, or incomplete?
Do you recognize when tics are about to happen?
If you have any such sensations or you can recognize before you do a tic, please answer the following questions.
Regarding the items below, how much does each one fit the everyday you?
Put a circle on the number that you think best fits.

0 Not at all true
1 Not true
2 Very slightly true
3 Somewhat true
4 True
5 Very True

１. After the sensation emerges, I'm likely to be bothered by it repeatedly, again and again.	0	1	2	3	4	5
２. When the tic is about to appear, I can often notice it.	0	1	2	3	4	5
３. In some cases, I don't notice that the tic has appeared.	0	1	2	3	4	5
４. Once the sensation has emerged, I will just keep being bothered by it.	0	1	2	3	4	5
５. Before I experience a tic, I know what kind of tic it will be.	0	1	2	3	4	5
６. Sometimes, I focus on the sensation for dozens of minutes.	0	1	2	3	4	5
７. I often experience a tic without noticing it.	0	1	2	3	4	5
８. I feel a sensation several times a day, but it does not bother me for a long time.	0	1	2	3	4	5
９. I experience more tics with a prior sensation than I do tics without warning,	0	1	2	3	4	5
１０. Sometimes, I feel bothered by a sensation all day long.	0	1	2	3	4	5
１１. Often, I feel some kind of sensation before the tic appears.	0	1	2	3	4	5
１２. Sometimes, I just keep feeling bothered by the sensation for over 30 minutes without a break.	0	1	2	3	4	5
１３. I notice tics before they appear, tics do not happen automatically.	0	1	2	3	4	5
１４. Often, I just can't stop focusing on a sensation.	0	1	2	3	4	5

Tic Suppression Scale

We will ask about how you normally deal with tics (e.g. suppressing them to keep them from appearing or using strategies to ensure they are not conspicuous). Read the following sentences and put a circle on the number that you think best fits.

1. How often you suppress the tic in situations where you don't want the tic to be conspicuous?	0. Rarely<br>1. Sometimes<br>2. Often<br>3. Always
2. How often do you suppress the tic when the tic exhausts or injures you?	0. Rarely<br>1. Sometimes<br>2. Often<br>3. Always
3. How often do you suppress a tic that disturbs daily behavior or conversation?	0. Rarely<br>1. Sometimes<br>2. Often<br>3. Always
4. Have you ever suppressed a tic before? If you have never once thought to suppress a tic, circle on "1. No" and advance to 14. If you have tried to suppress a tic, continue from 5 onward.	0. Yes<br>1. No<br>2. Unsure
5. What happens to your tic when you try to suppress it?<br>0. I cannot suppress it even though I want to.<br>1. I manage to suppress it slightly, but become unable to suppress it immediately after.<br>2. I manage to suppress it to some extent, but eventually become unable to suppress it.<br>3. I can keep it suppressed for a while.	
6. When you manage to suppress the tic, how long can you suppress it for?	0. Less than 1 minute<br>1. Some minutes to 10 minutes<br>2. Several 10 minutes<br>3. Over 1 hour
7. When you try to suppress a tic, do you always manage to do so? Does it vary between each time?	0. Always cannot suppress<br>1. Can sometimes suppress<br>2. Often can suppress<br>3. Always can suppress

8． When you try to suppress a tic, to what extent can you suppress it?

(If normal tic is 10, how much does size or strength reduce?)

0． Cannot suppress at all (tic's size and strength: 10 ⇒ Remains at 10)

1 . Can slightly suppress (tic's size and strength: 10 ⇒ Down to 7-9)

2． Can suppress (tic's size and strength: 10 ⇒ Down to 3-6)

3． Can largely/mostly suppress (tic's size and strength: 10 ⇒ Down to 0-2)

9． When you try to suppress a tic, what do you think happens to the tic as viewed by people around you?

0 . It is just the same as when I don't try to suppress it.

1 . It becomes slightly less conspicuous.

2 . It becomes not very conspicuous.

3 . un-noticeable.

１０． Normally, when you try to suppress a tic, to what extent do you feel you succeed in doing so?

0 . Don't succeed

1 . Slightly succeed

2 . Succeed to some extent

3 . Succeed to considerable extent.

１１． When you suppress a tic, how much do you experience a strange or insufficient feeling, or an unpleasant feeling, compared to when you produce the tic?

0 . Don't at all

1 . Do slightly

2 . Do

3 . Do severely

１２． How much effort or concentration power is required for you to suppress a tic?

0 . Scarcely required

1 . Slightly required

2 . Required to some extent

3 . Required to considerable extent

１３． Does your body ever get exhausted after you have suppressed a tic for a while or have tried to suppress it?

0 . Hardly ever get exhausted

1 . Get slightly exhausted

2 . Get exhausted to some extent

3 . Get exhausted to considerable extent

１４. How satisfactorily do you deal with your tic at present?

Put a mark (vertical line) somewhere between 0 and 100 along the line below, with 100 indicating case where you deal adequately with tic and are satisfied.

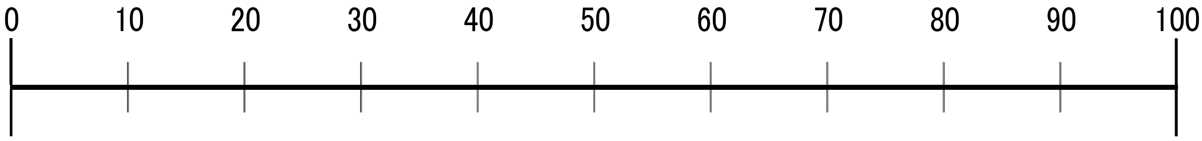

Severely dissatisfied with how I deal with tics

Line example

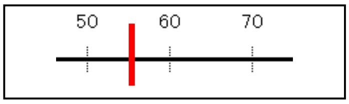

I deal with tics adequately and satisfactorally
